# Maintaining Cyber Resilience in the Reconfigurable Networks with Immunization and Improved Network Game Methods

**DOI:** 10.3390/s24227116

**Published:** 2024-11-05

**Authors:** Maxim Kalinin, Evgeny Pavlenko, Georgij Gavva, Maxim Pakhomov

**Affiliations:** Institute of Computer Science and Cybersecurity, Peter the Great St. Petersburg Polytechnic University, 29 Polytekhnicheskaya ul., 195251 St. Petersburg, Russia; pavlenko@ibks.spbstu.ru (E.P.); gavva@ibks.spbstu.ru (G.G.); pakhomov@ibks.spbstu.ru (M.P.)

**Keywords:** cyber resilience, immunization, network game, path length, reconfigurable network, security, topology

## Abstract

The paper proposes a technique for protecting reconfigurable networks that implements topology rebuilding, which combines immunization and network gaming methods, as a solution for maintaining cyber resilience. Immunization presumes an adaptive set of protective reconfigurations destined to ensure the functioning of a network. It is a protective reconfiguration aimed to preserve/increase the functional quality of the system. Network nodes and edges are adaptively reorganized to counteract an invasion. This is a functional component of cyber resilience. It can be implemented as a global strategy, using knowledge of the whole network structure, or a local strategy that only works with a certain part of a network. A formal description of global and local immune strategies based on hierarchical and peer-to-peer network topologies is presented. A network game is a kind of the well-defined game model in which each situation generates a specific network, and the payoff function is calculated based on the constructed networks. A network game is proposed for analyzing a network topology. This model allows quickly identifying nodes that require disconnection or replacement when a cyber attack occurs, and understanding which network sectors might be affected by an attack. The gaming method keeps the network topology resistant to unnecessary connections. This is a structural component of cyber resilience. The basic network game method has been improved by using the criterion of maximum possible path length to reduce the number of reconfigurations. Network optimization works together with immunization to preserve the structural integrity of the network. In an experimental study, the proposed method demonstrated its effectiveness in maintaining system quality within given functional limits and reducing the cost of system protective restructuring.

## 1. Introduction

Protection of reconfigurable networks (Internet of Things, industrial cyber-physical systems, wireless sensor networks, VANET/FANET networks, etc.) is becoming increasingly important due to their active utilization in various technological and civic areas such as smart homes, smart buildings, intelligent industrial environments, digital hospitals and smart power grids. Ensuring ubiquitous security, stable connectivity and permanent functional sustainability of these systems has become a top priority for both businesses and individuals, as the current landscape of possible cyber threats (e.g., denial of service attacks [1], node compromise attacks [2], man-in-the-middle attacks [3], and interference use [4]) requires quick response and adaptation to attacks.

To protect these networks and counteract cyber threats, recent protective techniques include various attack and intrusion detection [5,6,7,8,9], anomaly detection [10], and access control [11]. However, these methods protect individual nodes, and it is important to maintain the cyber resilience of a network which is the ability of connected functional systems to maintain normal operating under cyber attacks, preserve its optimal topology, and avoid node interference.

Cyber resilience is vital for reconfigurable networks because it allows:

- online security management and attack resistance. Optimal and stable distribution of nodes for information communication and the presence of backup channels allows effective inspection of network traffic [12], detection of anomalies and functional faults, and faster structural reaction to cyber incidents;
- interference reduction. Proper distribution of nodes avoids or minimizes mutual interference between nodes and neighboring networks, increasing the stability and security of communication;
- elimination of weak spots. If too many nodes in a wireless reconfigurable network are located in the same area close to each other, this can lead to channel interference, channel overload, data interception, and the implementation of near attacks. An optimal topology allows it to reduce the number of weak spots that can be used to implement attacks;
- performance optimization. As many spatial connection can be established in a reconfigurable network, the system can be overloaded by unnecessary links organized between nodes. This hardens the dynamic routing, overloads the inner structure of the system, supplies new possible attacks, and, as a result, slows down the system reaction to cyber threats.

The topic-relative approaches, such as network topology virtualization (e.g., [13]) and set-theoretic methods (e.g., [14]), cannot help us maintain the cyber resilience of reconfigurable networks. Network virtualization does not contribute to the flexible system resistance to security incidents. It only allows us to create a honeypot topology that imitates the real one for an adversary, but it does not allow to detect or prevent attacks. The set-theoretic approach is resource-intensive when dealing with constantly changing network topologies. It also does not support large-scale networks and is very slow and inert, not allowing online adaptation to changes in the number of nodes affected by attacks.

Because of high variability of a reconfigurable network, it is impossible to create an universal protective method. Hence, the proposed solution to this problem is a mechanism for dynamic system management based on two methods:

- immunization. This is a protective reconfiguration aimed to preserve/increase the functional quality of the system. Network nodes and edges are adaptively reorganized to counteract an invasion. This is a functional component of cyber resilience;
- optimal distribution of network nodes. This keeps the network topology resistant to unnecessary connections. The less disordered paths in the network graph, the more stable the system is. This is a structural component of cyber resilience.

Immunization presumes an adaptive set of protective reconfiguring measures destined to ensure the functioning of a network. It can be implemented as a global strategy, using knowledge of the whole network structure, or a local strategy that only works with a certain part of a network in the absence of knowledge of the whole network. Basic immunization strategies (e.g., [15,16,17]) are based on a centrality metric that can only be applied to hierarchical network structures. And it is commonly implemented by removing an injured node from the network structure. To support reconfigurable networks, system variability, and peer-to-peer structures, the immunization mechanism is proposed to be universalized by transforming it to an adjustable set of protective actions aimed at maintaining/improving the functional quality of the system.

The structure-oriented side of cyber resilience – optimization of network node distribution – brings us to a game modeling. A network game is a kind of the well-defined game model in which each situation generates a specific network, and the payoff function is calculated based on the constructed networks [18]. A network game is proposed for analyzing a network topology, where the modeling graph vertices represent the real network nodes, and arcs represent connections between real nodes. This model allows quickly identifying nodes that require disconnection or replacement when a cyber attack occurs, and understanding which network sectors might be affected by an attack. However, multiple topology changes during the game can, in some cases, lead to disconnections with non-compromised nodes, and overload the channels of control nodes, which may lead to exhaustion of node resources, and, consequently, loss of the resilience and functional integrity of the whole network. Therefore, it is suggested to improve the basic network gaming strategy in order to solve these issues and make rapid decisions on choosing a scenario for network reconfiguration.

To present the suggested cyber recilence maintaining technique, the rest of the paper is organized as follows. Section 2 presents the functional (immunization-oriented) and structural (improved network game-oriented) methods to protective reconfiguration of a network; Section 3 shows the results of an experimental study, demonstrating the effectiveness of the proposed methods; Section 4 summarizes our work and compares it with related studies; and, finally, Section 5 concludes this research and sets the further course.

## 2. Materials and Methods

### 2.1. Immunization Strategies

Due to the ongoing growth in the number of cyber attacks on reconfigurable networks and the expansion of the range of possible destructive effects implemented by adversaries, the task of equipping reconfigurable networks with cyber resilience property is especially relevant. This can be done at the system design stage, but due to the need to protect the already functioning system, there is a need to develop a mechanism that enhances system resilience under attacks. Because of the high variability of reconfigurable networks, it is hard to develop a single universal protective method. To address this issue, the reconfigurable networks are classified according to their infrastructure type, based on criteria such as object variability and vulnerability to cyber attacks:

- static hierarchical topology;
- static peer-to-peer topology;
- moving hierarchical topology;
- moving peer-to-peer topology.

In reference to the topology type, various immunization strategies can be observed:

- global immunization strategies using knowledge of the overall structure of the network;
- local immunization strategies based on the local information of the network domains.

The global immunization strategy, which requires knowledge of the entire network, is ineffective for networks with dynamic topologies whose nodes can move. The local strategy can be successfully applied if there is no complete information about the entire network, or if the network perimeter is changeable and, therefore, undefined. Immunization strategies typically target individual nodes or hubs to increase their level of protection and reduce the likelihood of cyber attacks occurring in the network. Under these conditions, the formalization of the problem of creating an immunization looks like as follows.

A reconfigurable network is defined as a system Sys characterized by a set of functional components (nodes) $Nodes=\left\{Node_{1},Node_{2},\ldots ,Node_{n}\right\}$, connections Edges between nodes, a type Type of network infrastructure, and a target function *F*: $Sys=\langle Nodes,Edges,Type,F\rangle$.

The function *p* defines the parameters of the nodes and connections that are valuable for the functional task being solved by this system: $p\left(Nodes\right)=\left\{p_{1}^{Nodes},p_{2}^{Nodes},\ldots ,p_{N}^{Nodes}\right\}$ and $p\left(Edges\right)=\left\{p_{1}^{Edges},p_{2}^{Edges},\ldots ,p_{M}^{Edges}\right\}$.

The function *q* defines the quality of realization by the system Sys of its target function: $q\left(F\right)=Q,\;Q\in \left[Q_{min};Q_{max}\right]$. $Q_{min}$ is the minimum acceptable value of the system work quality indicator. $Q_{max}$ is the highest level of the system quality.

Throughout its life cycle, Sys is injured by various cyber attacks. Let $Z=\left\{z_{1},\ldots ,z_{L}\right\}$ defines the set of attacks subjected on the system. Each attack $z_{i}\in Z$ is characterized by the number of destroyed system objects (nodes or edges) and a decrease of the quality *Q*. The impact of cyber attacks on a set of system objects is specified by the function z(t).

To counteract cyber attacks, a reconfigurable network uses an immunization mechanism *I*, which includes the security tools embedded in the system at the design time or added (installed) later during operation. Immunization implements global $I^{G}$ and local $I^{L}$ protective strategies: $I=\left\{I^{G},I^{L}\right\}$. Global strategy operates on the sets Nodes, Edges, and the quality *Q*; and local strategy – on the sets Nodes, Edges, $\left\{p_{1}^{Nodes},\;p_{2}^{Nodes},\ldots ,p_{N}^{Nodes}\right\}$, $\left\{p_{1}^{Edges},p_{2}^{Edges},\ldots ,p_{M}^{Edges}\right\}$ and the quality *Q*. Formally, the process of system immunization is expressed as a function of time I(t), which represents the recovery of system components damaged by attacks and the increase of the quality indicator *Q* decreased by attacks.

The immunization task is to minimize the number of the damaged network nodes and maximize the number of the cured nodes and the value of *Q*. Generally, the immunization can be specified as an operator Ψ following the conditions:

- Ψ:Z(t)−I(t)→min: minimizing the number of the injured nodes;
- $\Psi :I\left(t\right)\to Nodes_{0}-Z\left(t\right)$: maximizing the number of cured nodes, where $Nodes_{0}$ is the number of nodes before the attack;
- $\Psi :\left|Q_{0}-Q^{\prime }\right|\to min$: maximizing the value of *Q*, or minimizing the difference between the initial value $Q_{0}$ ($Q_{0}\in \left[Q_{min};Q_{max}\right]$) and the value of $Q^{\prime }$ obtained as a result of the attack.

For hierarchical systems, it is proposed to implement the immunization by the following steps:

1. Calculating the current value of a quality metric $Q_{0},\;Q_{0}\in \left[Q_{min};Q_{max}\right]$.
2. Calculating the metrics:
  centrality. It is calculated in accordance with the PageRank algorithm [19,20], designated $P\left(Node_{i}\right)$ and based on the importance of a vertex. The more important the vertex is, the more possible paths from all vertices of the graph to a given one:
  $P\left(Node_{i}\right)=d\sum_{Node_{i}\neq Node_{j}}a_{Node_{i},Node_{j}}\frac{P\cdot Node_{j}}{O\left(Node_{j}\right)}+\frac{1-d}{n}$, where $d\in \left[0,1\right]$ is an attenuation coefficient, usually taken to be 0.85, and $a_{Node_{i},Node_{j}}$ is an element of the adjacency matrix of the graph simulating Sys,
  $$a_{Node_{i},Node_{j}}=\left\{\begin{array}{c} 1,\;\left(Node_{j},Node_{i}\right)\in Edges\\ 0,\;\left(Node_{j},Node_{i}\right)\notin Edges \end{array}\right.;$$
  criticality by connectivity. It is denoted by $C\left(Node_{i}\right)$, where a node is critical if its removal will break the graph $G\left(s,d\right)$, where $G\left(s,d\right)$ are successful message transmissions from the source *s* to the recipient *d*.
3. Identification of a set of critical nodes $Nodes_{cr},\;Nodes_{cr}\subseteq Nodes$ by sorting the nodes and selecting nodes with the largest metric values of centrality or criticality introduced above in the step 2.
4. Application the global immunization strategies to nodes $Nodes_{cr}$:
  4.1. Removal of the component $Node_{i},Node_{i}\in Nodes_{cr}$.
  4.2. Removal of the component $Node_{i}$, and replacing it with a backup node ${{\mathrm{Node}}_{i}}^{\prime }$ such that $p\left(Node_{i}\right)=p\left({{\mathrm{Node}}_{i}}^{\prime }\right)$.
  4.3. Removal of the component $Node_{i}$, and distribution of edges incident to the node $Node_{i}$ between the nodes $\left\{Node_{j},\ldots ,Node_{k}\right\}\in Sys$ already functioning in the system.
  4.4. Creation of an alternative communication between the nodes $\left\{Node_{j},\ldots ,Node_{k}\right\}$ (in case of impact on the data transmission process) and construction of additional edges between this pair of nodes.

The changes being introduced are structural and require, like any global strategy, apriori knowledge of the network structure.

In peer-to-peer topology, the objective of an attacker is often to disable (or compromise) as many nodes as possible. Due to the absence of clearly defined critical nodes in a peer-to-peer network, the start of an attack can affect any node. In this regard, it is proposed to develop a local immunization strategy for peer-to-peer topologies based on a probabilistic approach.

Lets represent the graph modeling the system Sys as a random graph [21] with a degree distribution $P\left(x\right),\;P\left(\xi =x\right)$, that is, the vertex ξ has a degree *x*. Then $P\left(k\right)$ is a portion of nodes with a degree *k*.

We randomly select $N^{\prime }$ nodes from *N* nodes, where $N=\left|Nodes\right|$. The probability that a vertex of degree *k* will be selected for immunization is the ratio $\frac{k\cdot P\left(k\right)}{N\left(\sum_{k}k\cdot P\left(k\right)\right)}$, where $\sum_{k}k\cdot P\left(k\right)$ is the average degree of the graph vertices.

Considering the graph arc corresponding to the data transmission path over the network, we will select the levels. At the level *l*, there are $n_{l}\left(k\right)$ nodes of degree *k*. In next level l+1, each node will have degree k−1, minus this node at the previous level.

Let us denote by the event $A_{k}$ in which a node of degree *k* is a subject to attack from the set *Z* and is not yet immunized. To find the number of the attacked nodes $n_{l+1}\left(k^{\prime }\right)$ of degree *k*, we multiply the number of edges outgoing from level *l* by the probability of reaching the node of degree $k^{\prime }$ from a susceptible node: $p(k^{\prime }|k,A_{k})$. Then it is multiplied by the probability of that this node is also susceptible, taking into account both the degrees of the nodes and the fact that the neighbor node is also subject to attack: $p(A_{k^{\prime }}|k^{\prime },k,A_{k})$.

Then the number of nodes subjected to attack is calculated as follows:

$$n_{l+1}\left(k^{\prime }\right)=\sum_{k}n_{l}\left(k\right)\left(k-1\right)p\left(k^{\prime }|k,A_{k}\right)p\left(A_{k^{\prime }}|k^{\prime },k,A_{k}\right).$$

Using the Bayes’s formula, the probability that a node will be a subject to attack can be calculated as $p\left(k^{\prime }|k,A_{k}\right)=\frac{p\left(A_{k}|k,k^{\prime }\right)p\left(k^{\prime }|k\right)}{p(A_{k}|k)}$.

The number of nodes under the attack $n_{l+1}\left(k^{\prime }\right)$ can be considered as the number of nodes required for immunization. This parameter can vary depending on the density and connectivity of the network. The conditions for the occurrence of the event $A_{k}$ also depend on the attack type and the network properties, so it is advisable to set limits and determine the acceptable range for $n_{l+1}\left(k^{\prime }\right)$ on a case-by-case basis.

As a result, it is proposed to immunize a peer-to-peer topology according to a local strategy based on a lack of detailed information about the entire network and the probabilistic selection of a set of nodes to be immunized. The following steps are taken:

1. Calculating the current level of quality of the system $Q_{0},\;Q_{0}\in \left[Q_{min};Q_{max}\right]$.
2. Specifying the set of known attacks *Z*, to which the peer-to-peer network is subjected.
3. Defining the relationships between $\left(z_{i}\in Z,\;Nodes\right)$, characterizing the susceptibility of the system nodes to each type of attack $z_{i}\in Z$.
4. Specifying the probability of occurrence of an event $A_{k}$ for each $z_{i}\in Z$.
5. Calculating the corresponding probabilities and the number of nodes $n_{l+1}\left(k^{\prime }\right)$ to be immunized.
6. Immunizing $n_{l+1}\left(k^{\prime }\right)$ nodes by using one or more available actions:
  creating or activating a backup node for each one from $n_{l+1}\left(k^{\prime }\right)$ nodes;
  reducing the load on the node by increasing the charge level or signal quality, lowering the node degree, etc.

This algorithm is not exhaustive and can be expanded depending on the concrete system features and the real topology.

### 2.2. Improved Network Game Model

For the formal description of the proposed network game-based method for structural optimization in the reconfigurable networks, the following notation is introduced. A graph is a set $\langle N,E\rangle$ of vertices *N* and edges E⊆N×N. The set of arcs is the set of ordered pairs j∈E of elements i,j∈N. The incidence matrix $g=\langle N,E\rangle$ is used, which is an $\left|N\right|\times \left|N\right|$ matrix where $g_{ij}=1$ if *g* has an arc ij, and $g_{ij}=0$ if no.

$\Theta \left(N\right)$ is the set of all graphs with vertex set *N*. Following the classic network game theory, the interest of the *i*-th player in a particular connection structure is described by the payoff function $f_{i}:\Theta \left(N\right)\to R,i\in N$, which specifies the player’s payoff if certain structures are implemented. It is necessary to consider a situation where the consent of both players is required to establish a connection, such that trusted nodes in the system cannot accept connections from the possibly compromised nodes.

Then the intention of the *i*-th player to form a connection ij can be represented as a variable $x_{ij}^{out}$, which is equal to 1 if the player *i* wants to establish a connection ij, and 0 otherwise. Similarly, the variable $x_{ij}^{in}$ can be described, which indicates the *i*-th player’s consent to form a connection.

The profile of player actions in a network game is a pair $x=\left(x^{out},x^{in}\right)$ of square matrices of size *n*, the elements of which are the components of the allowed actions $x_{\mathrm{ij}}^{in}$ and $x_{\mathrm{ij}}^{\mathrm{out}}$ of players. The set of action profiles of a network game is denoted by $X=\prod_{i\in N}X_{i}$. The environment for the *i*-th player is a pair $x_{-i}=\left(x_{-i}^{\mathrm{out}},\;x_{-i}^{in}\right)$ of matrices of size $n\left(n-1\right)$, the elements of which are the components of the allowed actions $x_{ij}^{in}$, $x_{ij}^{out}$ of all players except the *i*-th player. The action profile *x* consists of the actions of the player $x_{i}$ and his environment $x_{-i}$, i.e., $x=\left(x_{i},x_{-i}\right)$.

If a certain action profile $x=\left(x^{out},x^{in}\right)$ has been implemented, then the resulting network *g* is determined by element-wise multiplication of the matrix $x^{out}$ by the transpose of the matrix $x^{in}$: $g=x^{out}⊕x^{in}$. The set of networks that can be obtained given a set of action profiles *X* is denoted by G(X).

The strategic model of a network game is a tuple $N,f_{i},X_{i},i\in N$ of a set of players *N*, their payoff functions $f_{i},i\in N$ and a set of allowed $X_{i}\subseteq X_{i}^{0},i\in N$.

The characteristic function is the Fourier transform of the distribution of a random variable $\Phi_{X}\left(t\right)=M\left[e^{itX}\right]$, where *M* is the mathematical expectation, the random variable is the payoff of each participant in the network game (discrete values). Then, assuming that the value of the payoff of the participants in the network game obeys a law of the form $\left(x_{k},p_{k}\right)$, where $p_{k}$ is the probability of winning $x_{k}$, the characteristic function has the form $\Phi_{X}\left(t\right)=M\left[e^{itX}\right]=\sum_{k}e^{itx_{k}}p_{k}$.

For the goal of self-regulation of a network infrastructure in accordance with the target function of the system, the gain of individual players is less important than the cost of the game. In the considered subject area, the cost of the game clearly shows the efficiency of the chosen scenario for self-regulation throughout the entire system while preserving the target function.

For an arbitrary network game $N,f_{i},X_{i}$, the cost function is the sum of the gain functions of all players: $v\left(g\right)=\sum_{i\in N}f_{i}\left(g\right)$, where $g\in G\;\left(X\right)$ is the network. The priority is to find distribution rules under which efficient networks are stable. For an arbitrary game N,v, a network $g\in \Theta \left(N\right)$ is efficient if the relation $g\prime \in \Theta \left(N\right),v\left(g\right)\geq v\left(g\prime \right)$ holds for any other network.

Agents (game players) participate in a network game $N,f_{i},X_{i}$. The gain of each player depends on which of the corresponding self-regulation scenarios that could counter the attack ($g\in G\left(X\right)$) has been implemented. The gain of the *i*-th agent is a function $f_{i}\left(\cdot \right)=f_{i}\left(g,u\right)$, where *u* is a certain control (motivation) implemented by the decision-making center among the set of permitted management variants *U*. The gain coefficient of the center depends on the control it has chosen: $\Phi \left(\cdot \right)=\Phi \left(g,u\right)$.

Let us imagine the order of operations of the considered system. According to this order, the method of dynamic reconfiguration of the network topology is implemented. The center provides control u∈U to agents. The agents use control to implement a network $g\in P\left(u\right)$ (where $P\left(u\right)\subseteq G\left(X\right)$). All participants in the system receive their gain: the center – $\Phi \left(g,u\right)$; the agents – $f_{i}\left(g,u\right),\;I\in N$.

The problem of managing the topology of a protected network in the context of self-reconfiguration comes down to choosing an acceptable control scenario in order to maximize the center’s gain, provided that, in case of fixed control, the agents (game players) choose a network based on the principle of rational behavior. In other words, in order to solve the problem of topology control, it is necessary to find $u^{*}\in Arg\max_{u\in U}\min_{g\in P\left(u\right)}\Phi \left(g,u\right)$.

In case of reconfiguring a network modeled by a graph, the center should strive to ensure that the agents form one of the many networks desirable for the center $D\subseteq \Theta \left(N\right)$ (according to the benevolence hypothesis). The target function of the center can be described as $\Phi \left(g,u\right)=\left\{\begin{array}{c} H_{1},\;g\in D\\ H_{2},\;g\notin D \end{array}\right.$, where $H_{1}\gg H_{2}$ is the gain of the center with favorable behavior of its agents; $H_{2}$ is the loss of the center with their unfavorable behavior.

At the same time, the target function of the center should contribute to maintaining the target function of the network. A center with such a target function is called a main center. For example, a center can strive to establish a fully connected network or, conversely, an empty network with no connections. It is advisable to consider a situation where the center exercises control over its own expenses with certain losses (the equivalent of these losses in the subject area could be the time spent reconfiguring the network or the quantitative value of a decrease in network throughput). Therefore, $\Phi \left(g,u\right)=H\left(g\right)-S\left(g,u\right)$, where *S* are the costs of network management.

A network game is a type of consensus game, i.e., to establish a connection between two players, both must agree to do so. The center with a payoff function $H\left(g\right)$, which depends on the set of connections formed between agents, can assign motivation functions $\sigma =(\sigma_{1}\left(g\right),\ldots ,\left(\sigma_{n}\left(g\right)\right)$ to agents to implement a particular scenario in the network. The center and agents receive complete information about the system parameters. The system has a standard operating procedure, in which the center makes the first step and informs the agents about a set of motivation functions that depend on their actions, then the agents choose actions to establish or break connections, and, at the final stage, the center collects income and stimulates the agents. In such conditions, the target function of the center is represented as the difference $\Phi \left(g,\sigma \right)=H\left(g\right)-\sum_{i\in N}\sigma_{i}\left(g\right)$, and the target functions of the agents are represented as the sum $f_{i}\left(g,\sigma \right)=f_{i}^{0}\left(g\right)+\sigma_{i}\left(g\right)$.

Since the center implements the benevolence hypothesis, the goal of the center is to create the following incentive system:

$$u^{*}=(\sigma_{1}\left(g\right),\ldots ,\left(\sigma_{n}\left(g\right)\right)\in Arg\max_{u\in U_{0}}\max_{g\in P_{1}\left(u\right)}\left[H\left(g\right)-\sum_{i\in N}\sigma_{i}\left(g\right)\right].$$

The solution is divided into two stages. At first, for each network $g^{*}$, a motivation system $\sigma^{*}\left(g,g^{*}\right)=\left(\sigma_{1}^{*}\left(g,g^{*}\right),\ldots ,\sigma_{n}^{*}\left(g,g^{*}\right)\right)$ is found that ensures the sustainability of the network $g^{*}$ with minimal costs $C\left(g^{*}\right)=\sum_{i\in N}\sigma_{i}\left(g^{*}\right)$ for the center. Secondly, the most profitable connection for the center is calculated in order to find the maximum expression $\tilde{\Phi }\left(g^{*}\right)=H\left(g^{*}\right)-C\left(g^{*}\right)\to \max_{g^{*}\in G\left(X\right)}$ in all possible networks $g^{*}\in G\left(X\right)$.

At a certain moment, a specific network node needs to exchange information with another node or group of nodes. In the absence of a direct link (arc in the graph), this node attempts to establish the required connection. Over time, without any restrictions, the graph of the network will increasingly tend towards a complete graph, which leads to a huge number of established but unused connections, as well as network overload and slow response to an attack. Therefore, it is necessary to limit the number of possible connections for end nodes, for example by using a central node [18]). As a limit to the network game, a rule can be added to control the maximum number of connections allowed per node, and this rule does not apply to the central node. If a node reaches the maximum number of connections and wants to establish another one, one of its previous connections will have to be broken.

This approach solves the problem of network congestion. However, if a large group of nodes has reached the maximum connection limit, even a simple action of adding one more node could cause a chain reaction of topology reconfigurations. To overcome this problem, a criterion for the maximum possible path length is proposed to improve the original network game method. When establishing a connection between two nodes, if there is already a shortest path between them (the path length is marked by *K*), then a direct connection will not be made and the flow will be directed along a shorter route via existing connections with adjacent nodes.

$d\left(i,j\right)$ denotes the distance between vertices *i* and *j* (i≠j), measured by the number of edges in the shortest path:

$$\left\{\begin{array}{c} d\in N,i↔j\left(\mathrm{nodes}\;\mathrm{are}\;\mathrm{reachable}\right)\\ d\notin N\left(\mathrm{nodes}\;\mathrm{are}\;\mathrm{unreachable}\right) \end{array}\right..$$

And, if vertex *i* needs to establish connection to vertex *j*, then:

$$\left\{\begin{array}{c} g_{ij}\to 1,\left(i↔j\;and\;d>K\right)\;or\;d\notin N\\ g_{ij}\to 0,\left(i↔j\;and\;d<K\right) \end{array}\right..$$

When a large number of nodes is reached, network segmentation is provided for the correct functioning of this method in each segment.

## 3. Results

The emulated reconfigurable network is an undirected graph, with vertices representing network nodes and edges representing connections between nodes. In addition to nodes that generate connection requests, there is a central node (vertex 0 in the graph model) that coordinates the network structure. The NetworkX library [22] was applied to build a model of the reconfigurable network topologies.

**Figure 1:**
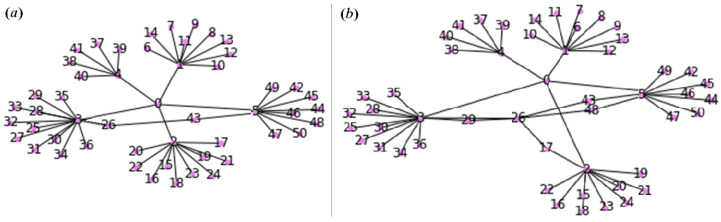
Sample of a hierarchical infrastructure: (**a**) a smart grid network; (**b**) result of the network immunization.

The experimental study of immunization is presented for the reconfigurable network with hierarchical structure. The hierarchical graph was modeled based on the system from [23] with a smart grid model [21], shown in Figure 1. This is a sample of a smart grid, an intelligent energy supply and consumption network. Figure 1a shows a network graph that simulates a smart grid segment with hierarchical structure, determined by types of devices: from sensors that perform only measuring functions to hubs that aggregate readings from sensors delivered using repeaters. The acceptable indicator of the quality of the system operation was data transfer efficiency, which varied in interval $\left[80\%;\;100\%\right]$.

During the experiments, an attack was simulated in the communication between a smart repeater and a hub. It was detected due to changes in the centrality metric values and a decrease in the data transfer rate between nodes. Global immunization strategies were implemented, which consisted of establishing additional connections between nodes (Figure 1b).

**Figure 2:**
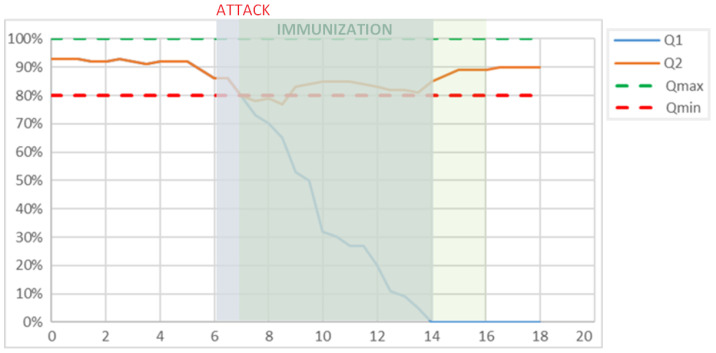
Demonstration of the immunization effectiveness.

The immunization trace is shown in Figure 2. The plot demonstrates that without immunization the system degrades very quickly. After the attack started at time point 6, by time point 8, the system was already operating with a quality parameter below the minimum level. By time point 14, the system failed. While the use of even the first immunization strategy already made it possible to counteract the cyber attack and maintain the system functional quality of the system within the specified range during the attack. The second strategy was applied at time point 12 when a decrease in the quality indicator started to be monitored and approached the minimum level of 80%.

**Figure 3:**
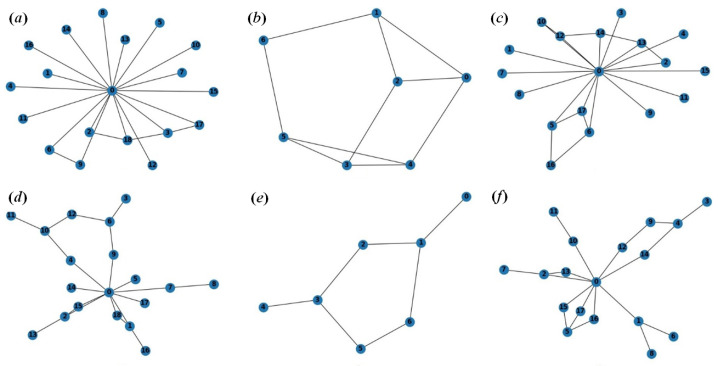
Experiments with the network game method: (**a**–**c**) original network game; (**d**–**f**) modified network game.

Protective immunization of the network leads to structural modification. Uncontrolled growth of the network topology may cause additional problems. So, network optimization works together with immunization to preserve the structural integrity of the network. During the experiments with the network structure optimization, three scenarios of topology reconfiguration were tested to assess the efficiency of the developed topology management method based on the improved network game (Figure 3):

- broadcast requests (Figure 3a,d). When establishing a network, requests are used that include several requesting and requested vertices for connection in each task;
- sequential connections (Figure 3b,e). When establishing a network, simple requests are used that involve sequential information exchange between nodes;
- mixed requests (Figure 3c,f). When establishing a network, both broadcast and simple requests are used that involve sequential information exchange between nodes, as well as requests for removing the vertices.

**Figure 4:**
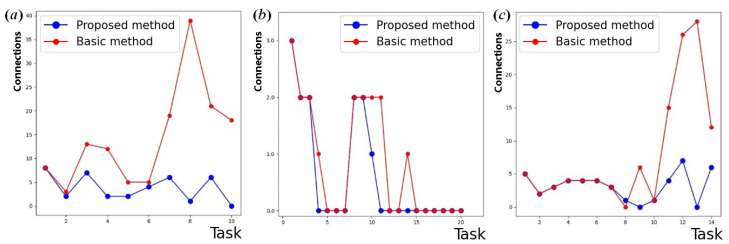
Results for direct connections: (**a**) broadcast; (**b**) sequential; (**c**) mixed requests.

**Figure 5:**
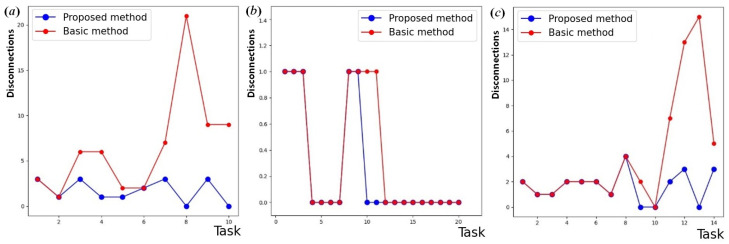
Results for direct disconnections: (**a**) broadcast; (**b**) sequential; (**c**) mixed requests.

When establishing the connection dynamically up to a certain time, the differences between topological pairs (according to the original and implemented methods) are almost not visible. However, when connecting to existing nodes with a large number of affected vertices in a query, these differences become crucial. To monitor this observation, we calculated the numbers of direct connections formed and broken at each step. Figure 4 shows the time diagrams of direct connections for broadcast requests, sequential requests, and mixed requests. The same scenario for node disconnections is plotted in Figure 5.

**Figure 6:**
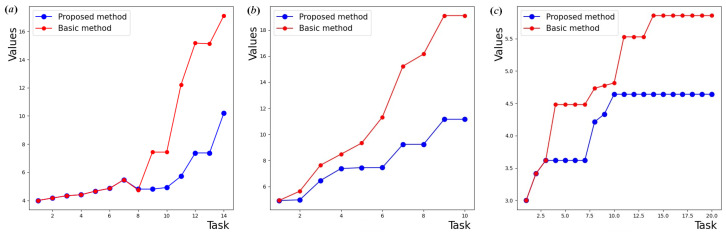
Results for spectral radius of the graph: (**a**) broadcast; (**b**) sequential; (**c**) mixed requests.

The spectral radius of the graph’s Laplacian is chosen as an additional indicator for quality measuring of the developed method. For the graph incident matrix $g=\langle N,E\rangle$, the graph’s Laplacian $L=\langle D-g\rangle$ is calculated, where *D* is a diagonal matrix of degrees of vertices (the diagonal elements of the matrix *D* are equal to the number of edges coming from each vertex). The eigenvalues of the Laplacian are then calculated: det(L−λI)=0, where *I* is a unit matrix and λ is eigenvalues. The spectral radius $\lambda_{max}\left(L\right)$ is the largest eigenvalue of the Laplacian. It gives information about the connectivity and stability of the graph. Spectral radius values under three test scenarios are shown in Figure 6.

As follows from this experiment, the first few values, regardless of the game method used—original or improved, are almost similar. This illustrates the small number of nodes in the reconfigurable network and the low degree of connectivity. The number of reconfigurations initiated in the network topology when using the original network game method is always higher than in the improved network game method. When using the proposed method with path length limit, a connection request does not require network reconfiguration, which indicates a network resistance to various types of structure rebuilding.

As shown in the figures, the difference in network topology is least noticeable when forming the sequential connections, as there is a high probability of appearance of a node having a direct connection already organized in the existing topology. The number of direct connections and disconnections in the network topology applied with the original network game method depends directly on the complexity of the topology. In the improved network game method, these characteristics are more influenced by the current configuration.

The spectral radius of the graph Laplacian when constructed according to the developed method is numerically smaller or equal to the spectral radius of the graph Laplacian according to the basic network game method. This indicates that the network topology is more resistant to targeted attacks on critical nodes, reduces the speed of malware propagation, and complicates the task of finding a controlling node for an intruder. At the same time, the spectral radius is maintained at a sufficient level, which indicates the presence of redundant paths in the graph, reducing the probability of network rupture in case of accidental failure of one of the nodes.

## 4. Discussion

A comparative analysis of the relative approaches to protecting reconfigurable networks that implement topology management allows us to identify the following areas of research (Table 1):

- network topology virtualization (e.g., [13,23,24,25,26,27,28]). An intruder reads the virtual topology of a network. This complicates isolating traps, breaking connections, and implementing denial-of-service attacks;
- set-theoretic approach based on network state modeling (e.g., [14,29,30,31,32,33,34,35]). The model of the system states, current security policy constraints, and cyber threats are performed. The model is used to predict changes in topology caused by node movement and reconnection. If security policy constraints are violated, counteractions are generated based on the model. Counteraction is a modeled sequence of actions to avoid security failure. Various variants of counteraction constructed in the model are applied to calculate adaptation strategies for a certain number of subsequent topological states of an under-controlled system;
- immunization. Networks play a crucial role in many diverse systems. Connectivity of components is critical for maintaining the functioning of large distributed structures as well as for developing efficient immunization against cyber threats. Because of this importance, researchers have focused on how a network can be optimally immunized to prevent intrusion or maintain infrastructure resilience. Many approaches have used reconfiguration and probabilistic methods to assess network resilience under the impact (e.g., [36,37,38,39]);
- game theory approach. It exploits the self-configurability of the reconfigurable network to help the system to maintain the target function by rebuilding the network structure. To ensure that a cyber attack does not disrupt the implementation of the target function, compromised or disabled nodes must be excluded or replaced with other nodes of similar functionality like in a game [18,40,41,42,43,44].

A network game is a type of game model where each situation generates its own network. The payoff function for this game is calculated based on the constructed networks [18]. A network game can be used to analyze reconfigurable networks, as it directly corresponds to a system model—graph vertices represent the network nodes, and the graph edges represent the connections between nodes. This allows us to quickly identify which nodes need to be disconnected/replaced when an attack occurs, as well as to understand which network sectors may be affected by an attack.

Virtualization allows us to create a false topology that differs from the real one, this hardens the adversarial activity in real network, but it does not react to attacks. The network debugging and path tracing allow an intruder to reconstruct the real topology [13]. The set-theoretic approach is not compatible with large-scale networks; it is very slow, inert, and expensive [14]. For a large-scale system, choosing an optimal reconfiguration scenario is quite difficult. Therefore, the most promising approaches are immunization and gaming.

Known immunization-targeted studies have shown that the network infrastructure of modern networks is variable and directly dependent on their complexity, structure and application scenario. Industrial and power plant networks are widely based on a hierarchical topology, implying a clear separation of system components based on their purpose and complexity. For smart monitoring networks (e.g., WSN, smart agriculture system, medical IoT), it is common to use a peer-to-peer topology, distinguished by the widespread use of close-related low-power nodes, the main purpose of which is to transmit aggregated monitoring data to a data processing center. Therefore, approaches to immunizing such networks vary significantly. Immunizing real networks against attacks is highly challenging due to the presence of hubs that prevent the eradication of an attack even if many nodes have been immunized.

For example, the studies [17,24,25] use centrality metrics for immunizing various types of networks, but this is only suitable for a well-known network structure, as well as its fixed hierarchical topology. Very important drawback of the related works is that they only concern the network centrality metrics, which is insufficient in case of systems integrating information and physical processes. For cyber-physical systems, in addition to centrality metrics, it is also required to concern of the parameters of network nodes associated with the physical processes performed by the system. In case of wireless networks, such important characteristics will be, for instance, the signal strength of a node and its battery charge level. Monitoring these characteristics in combination with centrality metrics will allow more accurate detection of attacks in the network and control of their influence on the system operation. Probabilistic approaches to immunization are also can be used for immunization of peer-to-peer networks [26]. Their advantage is that they usually do not require apriori knowledge of the entire network structure. But, unfortunately, there is no practical use model for the probabilistic immunization.

Network game is an approach that takes into account the flexibility of the modeled network and allows us to dynamically optimize the network structure. However, during the game play, the original network game method may cause disconnection of normal nodes and overload the networks leading to exhaustion of resources and loss of functional integrity of the entire network [45,46]. The proposed improvement to the network game method addresses these issues by implementing rapid decision-making on choosing a scenario for protective reconfiguration of the under-controlled network.

The developed cyber resilience technology provides the following advantages for maintaining the connectivity and sustainability of the reconfigurable networks:

- a backup route is guaranteed, along which traffic can be redirected to the destination node, even if an attack occurs in the system, and some channels are overloaded;
- system adaptability is provided when the size of the managed network increases. When connecting or reconnecting the nodes, a series of reconfigurations take place in a large network section. The developed technology ensures traffic is redirected through already established channels, preserving the system’s target function;
- the cost of reconnecting nodes is reduced due to continuous communication between the nodes;
- the impact on the target function of the managed network is reduced when compromised nodes (or network segments) are removed, thanks to the immunization effect and optimal route between adjacent nodes.

Used approaches were compared in deep (Table 2). The computing complexity of these methods is compared under unintentional network operation. For this purpose, the following notation is introduced: *N*—a number of nodes in the network, $n_{1}$—a number of nodes provoking communication, $n_{2}$—a number of nodes requested for communication, *q*—a number of access level rules, *z*—a number of states to be predicted, *h*—a number of state-to-state transitions of the system to reach a safe state.

## 5. Conclusions

The ability to dynamically rebuild the network topology determines the cyber resilience of the reconfigurable networks. An analysis of related methods for protecting these networks based on topology management has identified immunization and network game approaches as the most effective. The combination of these methods provides an adequate response to cyber attacks and topology changes by adapting the network management strategies.

The proposed immunization strategies are classified into global one that requires knowledge of a network structure, and local one based on transmitted data and individual node properties. This research describes an approach to network immunization using hierarchical and peer-to-peer topologies. In the first case, global immunization strategy is applied to network nodes with high criticality. Criticality is determined using centrality metrics and network connectivity parameters. In the second case, a probabilistic approach is used to select a certain number of nodes for which protective measures are applied.

The original network game model has several drawbacks: overloading of control node channels, excessive sensitivity to new tasks in topology when connecting and disconnecting nodes. To solve these problems, a maximum possible path length criterion was added to the basic network game strategy, reducing the number of network reconfigurations when a shorter path exists passing through existing connections.

An experimental study has demonstrated that using immunization during a cyber attack on a system allows it to maintain system functionality within given limits. The system is able to operate reliably when subjected to a cyber attack. Therefore, a positive effect has been achieved in ensuring the cyber resilience of complex networks.

Experiments with the developed network game-based method have confirmed that it is more reliable and effective than alternative approaches and the basic network game model. The proposed modification of the network game allows maintaining the functional resilience of reconfigurable networks online at lower costs and faster time for rebuilding network topology.

Our further research aims to establish the relationship between system stability and local immunization strategies in case of peer-to-peer network topologies. Also, our work will focus on the usecase application of the proposed solution for the IoT and Vehicle Adhoc NETworks (VANET), ensuring adaptive network security.

## Figures and Tables

**Table 1:** Comparison of the related approaches.

Criterion	Virtualization	Immunization	Set-Theoretic Approach	Game Theory
Flexible incident response (change of protection strategy when a node is compromised)	−^1^	+	+	+
Adapting the protection strategy when changing the network topology	+	+	+	+
Fault tolerance (maintaining network functionality after node failure)	+	+	+	+
Elasticity of the solution when the size of the managed network changes dynamically	May degrade	May degrade	−	+

^1^ ‘+’—feature is provided by method; ‘−’—feature is not provided by method.

**Table 2:** Comparison of the computational complexity.

Characteristic	Virtualization	Set-Theoretic Approach	Proposed Method
Computational complexity	$O(N^{2}n_{1})$	$O(Nn_{1}qzh)$	$O(n_{1}n_{2})$

## Data Availability

Data are contained within the article.
